# Reducing the risk of infection after total joint arthroplasty: preoperative optimization

**DOI:** 10.1186/s42836-019-0003-7

**Published:** 2019-08-01

**Authors:** Brielle Antonelli, Antonia F. Chen

**Affiliations:** Department of Orthopaedics, Brigham and Women’s Hospital, Harvard Medical School, 75 Francis Street, Boston, MA 02115 USA

## Abstract

Total joint arthroplasty (TJA) is one of the most commonly performed procedures in orthopedic surgery, and as the demand for TJA increases over time, the number of concurrent complications such as surgical infection will also increase. There are multiple risk factors that independently increase the risk of surgical site infection (SSI) and periprosthetic joint infection (PJI) after surgery. These modifiable risk factors can be identified in preoperative clinic screening visits that gives physicians the opportunity to provide specific intervention that can decrease patient infection risk. The risk factors that are known to significantly increase the risk of PJI and/or SSI include MSSA/MRSA colonization, rheumatoid arthritis, cardiovascular and renal disease, obesity, diabetes mellitus, hyperglycemia, anemia, malnutrition, tobacco use, alcohol consumption, depression, and anxiety. Patients who present with one or more of these risk factors require intervention with a multidisciplinary approach including patient education, counseling, and follow-up. Preoperative patient optimization for high risk TJA patients can significantly decrease PJI and SSI risk while improving surgical outcomes and patient care.

## Introduction

The number of total joint arthroplasties (TJAs) performed has increased over time, and the projected growth for total knee arthroplasty (TKA) and total hip arthroplasty (THA) from 2005 to 2030 is approximately 673 and 174%, respectively [1]. However, this growth in surgical procedures is associated with an increase in the number of surgical complications, such as periprosthetic joint infection (PJI). In order to reduce postoperative complications, infections should be prevented by engaging in patient optimization and targeted intervention of potential risk factors.

Patients’ risk for infection can be evaluated preoperatively with screening methods and transparent patient-provider communication, as patients undergoing TJA often have medical comorbidities or follow lifestyle activities that increase the risk for developing PJI and/or surgical site infection (SSI). The most common modifiable risk factors in 80% of eligible arthroplasty patients are obesity, anemia, malnutrition, and diabetes mellitus [2]. Other factors which warrant preoperative screening include methicillin sensitive *Staphylococcus aureus* (MSSA) and methicillin resistant *Staphylococcus aureus* (MRSA) colonization, rheumatoid arthritis, tobacco and alcohol use, renal failure, cardiovascular issues, depression, and medication use [3]. Some risk factors are more common in patients greater than 64 years of age, who often have longer hospital length of stay (LOS), which is an independent risk factor for PJI, and may have 5-times greater mortality compared to younger patients [4, 5].

Preoperative screening should occur at the initial visit, evaluating for factors such as obesity and diabetes, which may require longer intervention times. These preoperative clearance visits usually occur 2–6 weeks prior to surgery to allow adequate time for intervention and treatment, such as *Staphylococcus aureus* screening to permit sufficient time for the antibiotic treatment to be medically effective [6]. Each risk factor has an individual component that contributes to a heightened risk for SSI/PJI, and patients with more than one major risk factor or comorbidity are at an even greater risk for PJI development. These patients require intervention using a dynamic approach of risk factor recognition, medical treatment, education, and long-term engaged outlook to prevent risks from recurring. The purpose of this review is to identify specific modifiable risk factors that can be addressed preoperatively to optimize patients and reduce the risk of infection after TJA.

## MSSA/MRSA

Patients with pre-existing MSSA and/or MRSA colonization have an increased risk for developing postoperative orthopaedic SSI/PJI [7]. MRSA was the cause of 23% of primary THA SSIs and 21% revision THA SSIs [6]. These infected cases are difficult to treat and often result in unfavorable postoperative outcomes for patients, such as increased mortality and longer LOS [8]. Therefore, preoperative screening for MSSA/MRSA may be beneficial since *S. aureus* genotype screening shows that 80% of infections result from patients’ clonal nasal flora [9].

**Fig. 1:**
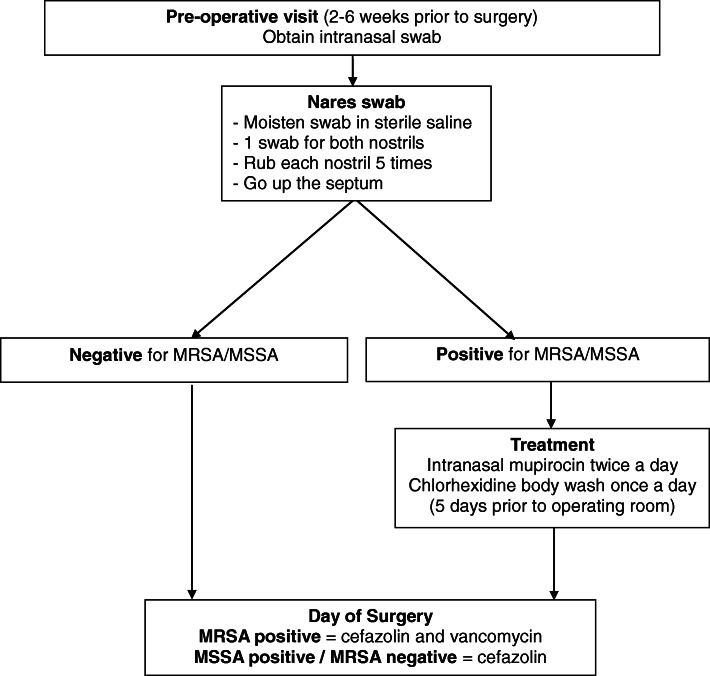
Methicillin sensitive and resistant *Staphylococcus aureus* screening regimen

Medical providers often perform MSSA/MRSA nares swabbing preoperatively in order to identify the presence of these organisms, although other sites can be swabbed including the axilla and perineum. Nasal swabbing successfully detected 66% of carriers, and overall detection rates reached 82% when both nosal and perineal swabbing were conducted [10–12]. If MSSA and/or MRSA is detected, the most common treatment involves the intranasal application of topical mupirocin ointment twice daily and 5 days of daily chlorhexidine body wash immediately prior to surgery [10, 13]. If a patient is MRSA positive, antibiotic prophylaxis with vancomycin is added to the usual regimen of cefazolin prior to surgery (Fig. 1).

MSSA/MRSA surveillance in patient populations has demonstrated that this intervention reduces infections and is cost-effective. Decolonization following nasal mupirocin application and antiseptic body washes decreased MRSA-related SSIs from 2.3 to 0.3% [14]. The cost of the nasal swab test and treatment ranges from $100 to $300 based on how many swabs are collected, insurance reimbursement, and decolonization regimen used. With decolonization rates exceeding 50%, the cost analysis of this regimen was deemed cost-effective and finically dominant from the hospital and third-party payer perspectives [15]. Success of this preoperative decolonization protocol was predicted in another study to save one hospital $230,000 [16]. The overall cost savings from avoided medical costs of treating infections and health benefits significantly outweighed the cost of MSSA/MRSA screening swabs and decolonization [15].

## Rheumatoid arthritis

The presence of rheumatoid disease is associated with a 3.7% increase in PJI which escalates with greater chronicity of rheumatoid disease [17]. This inflammatory disease impacts immune system function and adversely affects wound healing. The immunosuppression prescription medications often given to rheumatoid arthritis patients also contributes to increased infection risk [17]. Therefore, it is strongly recommended that preoperative clinic visits for patients with rheumatoid disease include education and guidance for modified medication regimens leading up to orthopaedic surgical procedures. These nonbiologic disease modifying antirheumatic drugs (DMARDs) should be discontinued before surgery due to their long half-lives and resulting elimination of underlying disease suppression [13]. Associated drug treatments often increase patients’ risk for developing mycobacterial and opportunistic infections [13]. Therefore, altered medication regimens include stopping biologics (i.e. rituximab and belimumab) before surgery and scheduling an arthroplasty date at the end of the dosing cycle for these specific medications. Patients should also be advised to stop the following medications at least 4 weeks before surgery: infliximab (REMICADE®), adalimumab (HUMIRA®), certolizumab (CIMZIA®) golimumab (SIMPONI®), and etanercept (ENBREL®), which should be stopped 15 days before the procedure [18]. At least 1 week before surgery, it should be noted that tofacitinib should also be stopped. These medications should only be started again after the healing process is complete with no signs of infection [3]. The medications that can be continued are methotrexate, leflunomide, hydroxychloroquine, sulfasalazine, mycophenolic acid (myfortic), azathioprine, mizoribine, cyclosporine, and/or prograf [19].

The use of corticosteroids to treat rheumatoid arthritis is another risk factor for infection where the degree of infection risk is directly proportional to steroid dosage [20]. These steroids increase the risk of infection due to depressed immune function with reduced phagocytosis, adhesion, leukocyte function, and vascular permeability [13]. Therefore, patients undergoing arthroplasty should ideally decrease the dosage of steroids prior to surgery to under 5 mg/day [21]. However, if this is not possible, the current daily dose of glucocorticoids should be given on the day of surgery if taking < 15 mg instead of giving perioperative supraphysiologic doses referred to as “stress doses” [19].

## Cardiovascular diseases

Cardiovascular disorders are linked to higher postoperative complications and PJI rates. Conditions such as myocardial infarction, atrial fibrillation [5], congestive heart failure [22], hypertension [23], pulmonary circulation disorders, peripheral valvular disease, and valvular disease are significant risk factors for PJI development [22]. TJA patients with atrial fibrillation also have an increased need for blood transfusion and prolonged LOS by approximately 3 days with higher complications and readmissions [24]. TKA patients with preexisting cardiac comorbidities have 16 times greater 30-day mortality compared to those without any cardiovascular conditions [25]. During the preoperative consultation, a complete cardiovascular history should be obtained to identify preexisting conditions that can increase the risk for postoperative complications. Some patients with prior cardiovascular issues scheduled for elective orthopaedic surgery should also be cleared by a cardiologist, as these screenings and interventions can reduce postoperative complications. These patients may benefit from undergoing echocardiography and subsequent optimization before undergoing surgery [26].

Anticoagulation medications for treating cardiovascular conditions, such as heparin, warfarin, or clopidogrel, have also been associated with increased PJI risk [24, 27]. These therapies can interfere with wound healing by causing complications such as hematoma, excessive wound drainage, and bleeding that can further predispose patients to PJI [27]. These anticoagulation regimens are often monitored using laboratory tests, such as international normalized ratio (INR), prothrombin (PT) and partial thromboplastin time (PTT). Higher preoperative INR levels have been associated with PJI in TJA patients [28]. INR levels above 1.5 among TJA aseptic revision patients were 2-times more likely to develop PJI [27]. Therefore, it is recommended that preoperative screening should include INR levels that should be <= 2 prior to surgery [29]. Patients on anticoagulation therapy may need to be seen at thrombosis clinics for proper therapy management and standardization of INR levels. These patients should also stop anticoagulation medications before surgery to decrease their risk of increased bleeding, wound complications, and infection. Some patients with myocardial infarction or atrial fibrillation may be able to change anticoagulation regimens and use protocols that may lead to less hematoma formation [5].

## Renal failure and dialysis

Renal system disease and renal failure patients are known to have greater surgical complications, such as infection, morbidity, and mortality after orthopaedic procedures [30, 31]. Multiple preoperative factors that predict the severity of renal disease progression can also highlight underlying, related comorbidities. Preoperative serum creatine level is an indicator of kidney problems and should be under 1.0 mg/dl [32]. The creatinine clearance formula predicts renal clearance in patients that takes body mass index (BMI) and age into consideration while measuring 24-h creatinine excretion [33]. Hemoglobin and potassium levels should also be checked since these patients often have anemia or hyperkalemia. If this is the case, preoperative hemoglobin should be corrected to > 10 g/dL and potassium < 5 mEq/L. [26] consultation with providers multiple providers is especially important to preoperatively correct and monitor fluid management, antibiotics, urea, glucose, and electrolyte levels in patients who often display imbalances [34]. Coexisting risk factors should also be taken into consideration and treatment plans are devised since factors such as nutrition/diet and lifestyle activities can individually predispose patients to increased infection risk with amplifying effects.

Dialysis patients have an even greater risk of postoperative infection, as these patients have weakened immune systems that slow the healing process [35]. TKA patients on dialysis have increased mortality (5.8%), early complications (58%), and deep infections (13%) [36] while THA patients on hemodialysis have 1-year mortality rates of 6.3% and patients with prior renal transplant also have high rates of mortality at 4.4% [37]. Overall, dialysis can increase the risk of infection up to 4-times compared to patients not on dialysis [31]. If medically feasible, dialysis patients may benefit from renal transplant prior to TJA to reduce their risk of infection [38]. However, if a patient is unable to receive a kidney transplant, dialysis techniques can be changed to potentially improve surgical outcomes by using bicarbonate-based dialysate and other biocompatible dialyzers [39]. Furthermore, infection rates are lower with preoperative hemodialysis compared to peritoneal dialysis, which is linked to higher amounts of skin and enteric flora [40]. Thus, preoperative consultation with nephrologists is recommended to reduce the risk of postoperative infection after altered dialysis regimens [31].

## Obesity

Obesity is another common modifiable risk factor for surgical infections, as almost 40% of the population is classified as obese with a body mass index (BMI) greater than or equal to 30 kg/m^2^ [41]. Obese orthopaedic surgery patients have doubled risk of developing a SSI [42]. Particularly for PJI, a prospective multi-site study reported that obese TKA patients were 6.7 times more likely to sustain a PJI while obese THA patients had a 4.2 times higher risk for developing a PJI [43].

Furthermore, surgeons experience challenging technical approaches, longer operating time, and more tissue dissection when performing surgery in obese patients. These patients are faced with a larger incision, more blood loss, slower healing, and longer recovery time that can make them more vulnerable to developing an SSI [10], prosthetic loosening, and the need for future revision surgery [44]. The operative time increases by 1 min for every extra 1 kg/m^2^ in body mass [45], which quickly accumulates for severely obese patients and put them in a very high infection risk. Postoperative outcomes are also jeopardized with increased pain and continued risk of osteoarthritis, as one pound of body weight equates to an extra four to six pounds of mechanical pressure on the knee joint [44].

The increased infection rates associated with obesity are also linked to the amount of adipose tissue which has higher bacterial counts [46]. Such characteristics of adpisoe tissue in obese patients are associated with altered surgical wound healing due to venous insufficiency [47, 48] and decreased vascularity [49], which lead to decreased oxygen supply to healing tissue and related oxidative stress [50], increased macrophage levels leading to acute inflammation [51], and other adverse healing effects. This is related to adipose tissue anatomy which can increase the amount of dead space in the wound and further delay healing [52]. Specifically, morbidly obese TKA patients (BMI < 40 kg/m^2^) had increased wound healing complications (22%) compared to normal BMI patients (2%) due to this increased dead space [53].

Other studies assessing adipose tissue properties have shown a relationship with higher counts of CD8 (+) effector T cells in epididymal adipose tissue, yet less regulatory T cells and CD4 (+) helper cells in obese mice [54]. This pathophysiological relationship with these immune cells in adipose tissue shows their influence on inflammation and decreased immunity associated with obesity [46]. Additionally, in obese THA patients, there were increased levels of cytokine mediators including interleukin (IL)-1 beta, IL-6, and tumor necrosis factor (TNF)-α levels. This is correlated to an increased postoperative proinflammatory state for obese patients that can lead to increased pain and slower recovery [55]. The expression of these inflammatory factors in peripheral blood lymphocytes have been notably imbalanced with obesity.

Therefore, the procedural BMI cut-off for elective TJA for most surgeons is 40 kg/m^2^ [56], while some surgeons may follow or are mandated to follow a cut-off of 35 kg/m^2^. Preoperative obesity screening should be implemented at least 6 weeks prior to surgery in order to give obese patients enough time to lose weight and improve their lifestyle before surgery in a safe and healthy manner. These patients should also have their glucose levels and blood counts tested, nutritional levels checked, along with cardiac and renal function assessed [56]. Patient education should also be included in the visit to inform patients of complications associated with obesity and the importance of losing weight before surgery. Providers may also refer patients to nutritionists and recommend exercise programs.

## Diabetes mellitus and hyperglycemia

TJA patients with blood sugar abnormalities are also at an increased risk for postoperative infections and other complications. Since the surgical process alters eating patterns and increases stress, this affects the body’s blood sugar response by increasing insulin resistance making diabetic and hyperglycemic patients especially vulnerable to adverse surgical outcomes [57].

### Diabetes mellitus

Pooled data for arthroplasty patients with diabetes in the U.S. displayed a significantly higher risk for SSI development with an odds ratio of 1.26 after the data *plural* were adjusted for hyperglycemia and BMI [58]. The odds ratio for development of an SSI was 2.8 [59]–3.4 for orthopaedic surgery patients with a preoperative serum glucose level of > 125 mg/dL or a postoperative level of > 200 mg/dL [60]. It has also been reported that diabetic TJA patients with unmanaged diabetes have a 2.8 higher PJI risk than non-diabetic patients [61].

Higher infection rates can be related to the decreased production of cytokines and formation of new blood vessels found at the wound site that make diabetic individuals more vulnerable to infection [62]. Furthermore, diabetic patients often have concomitant comorbidities associated with their condition, such as atherosclerosis that decreases proper wound healing, neuropathy that can lead to further musculoskeletal trauma, and vitamin D deficiency that can weaken bone [63]. Arthroplasty patients with diabetes treated with insulin have also been shown to have higher rates of 30-day readmission [64], longer LOS, and other renal, respiratory, and cardiac complications [65]. Multiple approaches have been implemented for optimizing diabetic patient health, such as preoperative screening for HbA1c levels (less than 7, 7.7 and 8%) and implementing glucose management programs to maintain healthy glycemic values and control.

### Hyperglycemia

In addition to diabetes, hyperglycemia increases infection rates due to its impact on the immune system and the healing process. This glycemic condition alters the role of leucocytes that can lead to an immunocompromised state and consequential deep tissue infection from surgery due to decreased innate immune function [66]. Since operating antagonizes insulin, surgery may predispose people to hyperglycemia which reduces the functional ability of leukocytes to fight infection [67, 68]. Arthroplasty patients who had hyperglycemic levels (> 137 mg/dL) the morning after surgery had an increased risk of developing PJI [69]. One study assessing primary TKA patients with perioperative glucose levels over 6.9 mmol/L displayed a 4-fold increase of PJI development compared to patients with normal glucose levels of < 6.1 mmol/L. [70] Accordingly, patients should have fasting blood sugar levels with or without the presence of ketones in their urine checked on the day of surgery [71]. Therefore, it is crucial for hyperglycemia to be managed before orthopedic surgery even though research is still exploring effective interventions in the perioperative and postoperative settings [57].

### Diabetes and hyperglycemia intervention

Preoperative identification of diabetic and hyperglycemic patients allows for proper intervention to optimize health before proceeding to surgery, which can significantly decrease SSI/PJI risk. Standard glucose monitoring and patient management should be implemented to keep glucose levels < 200 mg/dL and HgbA1C < 7% [72], which includes integrative care from primary care providers, endocrinology, rheumatology, internal medicine, and nutritional counseling with other educational outreach. Depending on a patient’s preoperative levels, surgeons will most likely suggest strict glycemic monitoring and control programs to help patients drop levels within the normal range; however, this can take some patients up to 6 months [73, 74]. Other alternative effective preoperative screening tools include glucose challenge tests (plasma or capillary glucose, GCTpl and GCTcap, respectively) and random plasma or capillary glucose (RPG or RCG respectively) [75]. Research has shown that GCTpl is the least expensive screening tool with subsequent effective screening results in high-risk patient populations [76]. The Medicare cost of using this screening tool over 3 years is $180,635 compared to the costs associated with no screening of $205,966 [75].

There have been successful outcomes with tight intraoperative glycemic control and the use of a basal bolus insulin regime that reduces rates of wound infection, bacterial counts, and acute respiratory and renal failure [77]. One study implementing an evidence-based approach to manage blood sugar in hyperglycemic patients effectively reduced SSI rates after THA and TKA [78]. Hyperglycemia and diabetes can also be treated with insulin after surgery, however, using a “sliding scale” with insulin correction is not routinely recommended as it can cause further complications and actually worsen hyperglycemia [57].

## Anemia

Anemia is classified by the World Health Organization (WHO) as hemoglobin levels less than 13 g/dL in men and less than 12 g/dL in women [79]. Anemia is detected in as many as 35% of elective orthopaedic surgery patients [80] and leads to increased LOS, infection, and mortality [81]. Research has identified preoperative anemia as an independent risk factor for PJI [82], as patients with low preoperative hemoglobin and hematocrit are more likely to receive transfusions and use anticoagulation medications that can also increase infection risk [3, 5, 83].

Patients with anemia should be referred to hematologists and other specialists to identify the cause of anemia in order to properly devise a treatment plan. Preoperative and intraoperative treatment has successfully reduced infection rates in anemic patients with the collaboration of surgeons, anesthesiologists, immunohaematologists, and other specialists [84]. Screening for anemia should be conducted around 30 days prior to surgery with blood work assessing complete blood count, iron (ferritin), vitamin B_12_ [85] and folic acid [86]. Causes may be malnutrition, chronic renal insufficiency, or chronic inflammatory disease [85]. For instance, if a patient is anemic due to low iron, physicians should not include administration of human erythropoietin (rHuEPO) because it will most likely lead to an adverse response to erythropoiesis and inhibit treatment [87].

For those with low iron contributing to their anemia, oral iron supplements (325 mg TID), vitamin B_12_ (1 mg), or folic acid (5 mg) taken 4 weeks before surgery can help improve patient’s laboratory levels before surgery to decrease their risk of infection [86]. This inexpensive and simple approach is recommended for patients who do not urgently need surgery. If oral iron cannot be tolerated, such as in elderly patients, intravenous iron can be administered since it  is faster-acting, safe, and has minimal side effects [84]. This treatment should work within 2–3 weeks to replenish iron levels and can raise hemoglobin levels by 1-3 g/dL after 1 month [88, 89]. Intravenous iron sucrose treatment has been proven especially beneficial in hip fracture patients which displayed less need for postoperative transfusions compared to patients without treatment [90]. Other intravenous iron formulations have also been effective in high doses with cost-effective benefits including ferric carboxymaltose, low molecular weight iron dextran, sodium ferric gluconate, or iron isomaltoside-1000 [84, 88]. If intravenous treatment does not result in normal hemoglobin levels within the expected time period, a dose of subcutaneous rHUEPO is advised [84].

Alternatively, preoperative erythropoietin helps to stimulate epoetin alpha (Epogen) which is a natural glycoprotein created by renal pericapillary cells in reaction to reduced oxygen tension, often found in conjunction with anemia or chronic obstructive pulmonary disease [91]. Epogen acts on bone marrow to stimulate red blood cell (RBC) differentiation and maturation, thereby increasing total RBC mass in anemic patients [91]. Its use has been approved by the Food and Drug Administration (FDA) in anemic patients with hemoglobin levels between 10 and 12 g/dL [92]. The use of erythropoietin is associated with decreased transfusion rates and consequently less PJI in TJA patients [93]. However, there are serious side effects with the administration of epoetin alpha, such as cardiovascular events, thromboembolic events, stroke, mortality, and tumor growth [94]. It is also expensive and can cost $3500 for a daily dose for 15 days and up to $2000 when administered weekly for 4 weeks [91]. Therefore, it is recommended that preoperative screening to identify the cause of anemia and eliciting respective alternative treatments should be considered before using Epogen.

The economic benefits of preoperative treatment for anemic patients undergoing elective orthopaedic surgery have revealed significant financial savings. Research assessing the economic costs associated with anemic orthopaedic surgery patients who followed preoperative treatment compared to those who did not reported a shortened LOS by 0.7 days and lower readmission rates by 5% that resulted in approximately $185,000 of savings [95]. Another large study was conducted by the Government of Ontario to assess the cost of implementing preoperative treatment programs for anemic patients undergoing cardiac and prostate surgeries, THA, and TKA. The programs costed $3 million to apply in practice, but healthcare savings reached $39 million with avoided surgical and postoperative complication costs [96]. Therefore, preoperative anemic screening with appropriate targeted treatment can significantly reduce SSI/PJI while also saving money.

## Malnutrition

Malnutrition often coexists with anemia and is another independent predisposing risk factor for orthopaedic surgical infections [97]. As many as 50% of orthopaedic surgery patients are malnourished and it is often not identified or treated preoperatively, which can lead to further complications [98]. Malnourishment can lead to suppressed immune responses, apathy, cardiac and renal complications [99], sarcopenia, hematoma formation [100], and impaired wound healing [101]. This is due to depleted protein reserves and inhibited proteoglycan and collagen synthesis which reduces wound healing capacity [10]. These various complications are especially prevalent in patients over 55 years of age undergoing TJA that often have other comorbidities that contribute to increased infection rates [100].

Nutrition markers can be checked easily with routine preoperative blood tests at least 2 weeks before surgery to identify at-risk patients and determine which metabolic markers are abnormal. Laboratory blood tests that indicate malnourishment include the following: albumin < 3.5 g/dL, prealbumin < 18 mg/dL, total protein < 6.0 g/dL, total lymphocyte count < 1500 cells/mm^3^, iron < 45 μg/dL, serum transferrin < 200 mg/dL, and 25-hydroxyvitamin D (25OHD) < 30 ng/mL [102]. Low albumin levels in THA patients were directly correlated to a 6-fold increase in 30-day mortality and major morbidity [103]. Low transferrin levels are also known to be an independent correlated risk factor for surgical infections [104] and predictive of delayed wound healing in THA patients [105]. Lymphocyte counts are often used as a nutrition marker even though studies relating them to wound healing and infection rates in orthopaedic surgery are not conclusive. Some studies have shown that low preoperative total lymphocyte counts were not predictive of malnutrition in some patients [106] and may not contribute to orthopaedic surgery infection [107] while others have shown that they did correlate with SSI [108], deep infection, excessive wound drainage [109], protein deletion associated with bone mineral density [110], and immunosuppressive effects [10] making patients vulnerable to infection.

Other significant markers are low iron, vitamin D, and total protein levels. Decreased iron levels are predictive of anemia and reduced protein counts that inhibit wound repair and overall healing [10]. Vitamin D is important for bone and muscle health, calcium regulation, and control of immune responses [111]. Low preoperative serum 25-hydroxyvitamin D (25OHD) in primary arthroplasty patients correlate with PJI and aseptic joint loosening [112]. Other preoperative screening tests for malnutrition incorporate alternative factors, such as preoperative total lymphocyte counts, albumin, transferrin, skin antigen testing, arm circumference, and triceps skin fold metrics. The results of these tests have been positively correlated to malnutrition status in orthopaedic patients [107]. TJA patients who were screened for malnutrition used similar integrated anthropoetric metrics such as the triceps skinfold, arm and calf muscle circumference, while also using standardized nutritional measurement tools, such as the Rainey-MacDonald nutritional index and the Mini Nutritional Assessment [102].

Malnourished patients should be given nutritional supplements daily for at least 14 days prior to their surgery date [113]. Specifically, diabetic and geriatric patients will need supplements that are low in sugar with strict glucose control, but also high in protein, vitamins and minerals.

## Smoking and alcohol use

Lifestyle factors including substance use, misuse and abuse further predispose TJA patients to increased SSI, morbidity, and mortality [114]. This is primarily due to their effects on wound healing and the introduction of foreign chemical materials into the body [115, 116]. These factors have detrimental physical and emotional or behavioral effects on patients who may be noncompliant to medical instructions and experience withdrawal effects resulting in other adverse outcomes [115]. Therefore, preoperative screening for lifestyle risk factors can help prevent infection development and may improve surgical outcomes.

### Tobacco use

Approximately 15.5% of American adults still report daily cigarette smoking even though the prevalence has decreased from 2005 to 2015 [117]. However, there has been no significant decrease in this rate from 2015 to 2016 [117]. The prevalence of smokers among TJA patients is also alarmingly high [118]. Cigarette smoking and the use of other tobacco products are known to have serious adverse health effects linked to the presence of 4000 hazardous chemicals, such as carbon monoxide, cyanide, nicotine, and other carcinogenic polycyclic aromatic hydrocarbons (PAHs) [119]. The health complications associated with these chemicals have also been shown to interfere with bone healing leading to reduced bone cell metabolic activity mostly attributable to nicotine [120], along with inhibited collagen synthesis and vasoconstriction which prolongs healing time [121], leads to wound necrosis, and weakens immune responses directly leading to infection [122]. This has been related to a decreased inflammatory response due to reduced immune cell chemotactic responses, oxidative bactericidal processes, and migratory ability [123]. Consequently, patients who smoke before surgery have significantly more postoperative complications and infection-related loosening with greater rates of revision surgery [114, 118]. This has led to increased national medical costs by $170 billion [124] and prolonged LOS by 4 days [125].

The direct association between smoking and PJI and consequential aseptic prosthetic loosing is a major concern for arthroplasty surgeons. For THA patients who smoked preoperatively, 1.5% of patients developed PJI with an overall 2.71-fold increased risk of postoperative infection [126]. This increased infection risk was also noted in TKA patients who smoke preoperatively [127] in whom smoking attributed to a 10.4% increase in SSI when calculated with a population-attributable fraction [128]. The frequency and length of time of smoking are influential when assessing patients’ infection and complication risk [129].

Another important factor which should be assessed when patients are seeking TJA is dental hygiene that is often negatively affected by tobacco use, alcohol consumption, and malnutrition. Tobacco use often results in poor dental hygiene that can influence infection risk after surgery [130]. Surgeons should make sure that patients do not have any ongoing dental infections or incomplete dental procedures before surgery and also do not present with decayed teeth, abscess, gingivitis, or periodontitis [72].

Since smoking is a modifiable risk factor, most researches suggest that smoking cessation programs should begin 6–8 weeks before surgery to effect changes [131], with a minimum time of 4 weeks [123, 132]. With a longer cessation period, postoperative complications are expected to decrease proportionally [133]. Orthopaedic surgery trauma patients following a 4 week smoking cessation program displayed reduced postoperative complication rates [129]. These patients displayed improved tissue oxygenation, inflammatory responses, and bone metabolism after smoking cessation for 4 weeks [123]. Some patients who may not follow such recommendations for an extended period of time can also abstain from smoking the day of surgery, which has shown less drastic but still significant reductions in SSI [134]. The most effective smoking cessation therapy programs have included a combination of weekly counseling sessions with a trained smoking cessation therapy nurse and nicotine replacement therapy at least 4 weeks preoperatively [135]. Other nicotine replacement therapies can include prescription medications such as buproprion SR (Zyban) or varenicline tartrate (Chantix), or over-the-counter products such as nasal sprays, nicotine patches, gum, and inhalers [136]. Smoking cessation can be verified with a simple serum cotinine test with a value ≤ 10 ng/dL [137], but it is important to note that even the use of nicotine patches will cause this test to be positive for nicotine.

This integrative approach has been proven to be cost-effective while also consequentially resulting in life-long health benefits if smoking is not continued postoperatively. More involved intensive therapy programs are cost-effective due to more significant net benefit with quality adjusted life year economic savings ranging from $1108- $4542 [138]. Patients who follow these programs and are persuaded to quit smoking preoperatively are at reduced risk for perioperative and postoperative complications that shortens their LOS and minimizes the need for additional surgery and treatment. Preoperative screening should include patient smoking history and the use of the Physician Quality Reporting System, which has been successful using physician-reported quality measures for Medicare [139] to reduce patient risk. Therefore, surgical patient optimization with preoperative screening, healthcare advising, and nicotine replacement treatment can significantly improve postoperative outcomes and reduce infection rates [140, 141].

### Alcohol consumption

Another modifiable lifestyle factor that is harmful to patients undergoing TJA is alcohol consumption. Patients with diagnosed alcohol abuse undergoing TJA have increased immediate postoperative complications such as stroke, surgical infections, blood clots, delirium, pneumonia, arrhythmia, gastrointestinal bleeding, and shock [142] along with longer LOS, and behavioral issues in TJA patients [116]. Excessive alcohol consumption is associated with organ dysfunction, cardiac insufficiency, varied hemostatic function, and immunosuppression that can be exacerbated in patients under increased surgical stress [143].

Chronic alcohol use increases an individual’s risk for additional medical problems due to its effect on the body. Long-term alcohol use predisposes patients to infection as it alters the immune system and T cell-mediated responses. Increased infection rates are further attributed to subdued cytotoxic lymphocyte ratio, inhibited interferon gamma:IL-10 ratio, and heightened levels of plasma IL-10 in alcoholics, suggesting overall suppression of whole blood cell responses [144]. Furthermore, among primary TJA patients, high alcohol consumption (> 168–252 g/week) was linked to a higher incidence of PJI at 1 year postoperatively compared to non-drinkers, low to moderate drinkers (> 0–168 g/week), or excessive drinkers (> 252 g/week) [145]. Excessive consumers displayed increased postoperative complications with deep venous thrombosis and increased 1 year mortality rates [145]. Researchers concluded that preoperative guidance and intervention for patients with low-to-moderate alcohol consumption can potentially be more lenient when suggesting abstinence, but preoperative abstinence should still be enforced for high and excessive drinkers [145].

Therefore, it is important to preoperatively screen every patient for a detailed alcohol history and quantify their usage and frequency. Screening measures used to identify at-risk patients include the Alcohol Use Disorders Identification Test (AUDIT-C), which is a self-reported questionnaire where each point on the 12-point scale attributed to alcohol consumption correlates to a 29% average increase in the number of surgical complications [142]. Healthcare professionals can also administer the Complications Evaluation Questionnaire (CEQ) to patients to assess the overall effects of alcohol and other lifestyle risk factors, such as tobacco [115]. Providers should also keep in mind that self-reported consumption levels can be underestimated and that alcohol abuse is defined as consuming at least 5 or more standard drinks per day [143].

Patients who follow professional alcohol cessation programs or cease alcohol intake have displayed improved reversal effects after abstinence. With alcohol cessation, organ dysfunction can be reversed over time and hemostasis can be improved within 4–8 weeks of alcohol abstinence [143]. After 3–4 weeks of abstinence, wound-healing capability is restored with significantly reduced postoperative morbidity and LOS [116]. Furthermore, within 1–2 months, cardiac and immune function can normalize, and external stress responses can be reduced after 3 months of alcohol cessation. This intervention should be multi-disciplinary and include counseling sessions, motivational health dialogue [143], pharmacological mediation, relapse prophylaxis with frequent follow-up, and medications if needed for withdrawal or alcohol substitution [146]. Such products that can be used include benzodiazepines for withdrawal [147], acamprosate [148], and opioid antagonists [149] for dependence, or disulfiram for short-term cessation [150].

Such an integrative preoperative approach for patients at risk can reduce postoperative complications and medical costs. Programs incorporating preoperative screening and counseling for patients have been proven cost-effective with medical savings around $1755 per quality adjusted life year [151]. These screening programs are important to identify preoperative alcohol misuse in TJA patients so that providers can intervene to implement effective multi-modal cessation programs to reduce infections and complications.

## Depression and anxiety

Lifestyle factors such as malnutrition, overeating, tobacco use, and alcohol consumption can be associated with altered emotional states and psychological conditions. Depression is a strong predictor of postoperative pain tolerance as it has been linked to decreased pain tolerance and increased postoperative infection and mortality [22, 152]. TKA patients with preoperative anxiety and/or depression were 6 times more likely to report dissatisfaction with long-term postoperative outcomes and had a longer LOS by 1 day compared to patients without either form of psychological distress [153]. Anxiety and depression often coexist and are both risk factors for TJA complications [154, 155], such as PJI [22, 156], due to the impact on the body’s immune response [157].

These adverse effects and increased infection rates can be explained by immunosuppression caused by depression leading to unregulated immune activation from inhibited T-cell activity [158] and affected serotonin pathways [152]. Genes in this pathway have been found with single nucleotide polymorphisms (SNPs) associated with depressive symptoms and unbalanced immune function linked to suppressed immunity and postoperative infections [152]. This condition leads to altered neurotrophic factor circulation and leukocyte responses [159]. Furthermore, a relationship between depression and inflammatory responses has also been identified. Also, higher levels of allelic variants have been expressed in depressed patients [160]. The etiology of depression has been linked to the expression of genes involving inflammatory molecules and enzymes. For instance, cytokines and enzymes involved in mediating the inflammatory response (cyclo-oxygenase2 [COX-2] and phospholipase2 [PLA2]) have been detected in depressed patients [152]. Other genetic variants are also associated with the biological mechanisms where depression develops from an altered innate immune system. This is linked to gene expression of IL-1β, TNF-α, C-reactive protein (CRP) and SNPs in the IL-1β, IL-6 and IL-11 genes that contribute to reduced efficacy of anti-depressant therapy [160]. Such altercations may prompt physicians and researchers to preoperatively detect these genetic variants in patients to assess their risk level. Due to these serious effects of depression presenting with altered immunomodulary responses that may lead to increased surgical complications and infection, depression and anxiety screening should be a routine part of preoperative assessments.

Screening for these emotional conditions is simple and can be conducted in clinics in a short amount of time. Common screening tools for depression and anxiety are the Patient Health Questionnaire-2 and -9 [152], the Hospital Anxiety and Depression Scale, and Beck’s Depression Inventory [161]. Preoperative screening and identification for the stage of depression is important because patients can also develop depression after surgery. Those with a history of preoperative depression are at a higher risk to relapse after surgery. The development of postoperative depression may be higher in orthopaedic surgery patients compared to other surgical specialties and can occur as soon as 2 days after surgery [162]. Preoperative evaluation and screening protocols should integrate and advise treatment from a psychologist or psychiatrist while specifically reviewing realistic patient expectations to avoid postoperative depression during recovery. Screening should also pay attention to the common comorbidities and lifestyle activities mentioned previously that often exist with depression and anxiety. Providers and counselors need to be patient with treatment and intervention outcomes, since most approaches for depression often fail or take a long time to produce positive results [152]. Preoperative screening and discussion with patients undergoing arthroplasty may reduce postoperative complications and PJI rates after appropriate intervention and therapy programs.

## Conclusion

In conclusion, all patients undergoing TJA are at risk for infection and complications, however, various predisposing modifiable risk factors have been identified that significantly increase SSI/PJI risk development. Medical comorbidities and lifestyle factors that independently increase one’s risk for SSI and/or PJI are MSSA/MRSA colonization, rheumatoid arthritis, cardiovascular and renal diseases, obesity, diabetes mellitus, hyperglycemia, anemia, malnutrition, tobacco use, alcohol consumption, depression, and anxiety. Comprehensive education and intervention are crucial to the optimization of TJA patients prior to surgery, as one study revealed that 80% of TJA cases had at least one modifiable risk factor present, with 46% of patients being obese, 29% anemic, 26% malnourished, and 20% diabetic [2]. Patients who were candidates for revision arthroplasty also require critical surveillance since researchers also reported that 93% of these patients had at least one modifiable risk factor present with anemia, urinary tract infection, and human immunodeficiency virus (HIV) being the most prevalent in this group, respectively [2]. Therefore, these reports support the need for preoperative patient screening to identify these risk factors before TJA along with the establishment of multi-disciplinary relationships with orthopaedic surgeons and respective care teams to improve patient outcomes. The resulting impact of implementing these preoperative protocols with widespread diligence and awareness may improve TJA infection prevention while providing satisfying patient care and surgical outcomes.

## Data Availability

Data sharing was not applicable to this article as no datasets were generated or analyzed during the current study.

## Abbreviations

25OHD: 25-hydroxyvitamin D
AUDIT-C: Alcohol use disorders identification test
BMI: Body Mass Index
CEQ: Complications Evaluation Questionnaire
COX-2: Cyclo-oxygenase-2
CRP: C-reactive protein
DMARDs: Disease modifying antirheumatic drugs
Epogen: Epoetin alpha
FDA: Food and Drug Administration
GCTcap: Capillary glucose
GCTpl: Plasma glucose
HIV: Human immunodeficiency virus
IL: Interleukin
INR: International normalized ratio
LOS: Length of stay
MRSA: Methicillin resistant *Staphylococcus aureus*
MSSA: Methicillin sensitive *Staphylococcus aureus*
PAHs: Polycyclic aromatic hydrocarbons
PJI: Periprosthetic joint infection
PLA2: Phospholipase 2
PT: Prothrombin
PTT: Partial thromboplastin time
RBC: Red blood cell
RCG: Random capillary glucose
rHuEPO: Human erythropoietin
RPG: Random plasma glucose
SNPs: Single nucleotide polymorphisms
SSI: Surgical site infection
THA: Total hip arthroplasty
TJA: Total joint arthroplasty
TKA: Total knee arthroplasty
TNF: Tumor necrosis factor
WHO: World Health Organization

## References

[1] Kurtz S, Ong K, Lau E, Mowat F, Halpern M (2007). Projections of primary and revision hip and knee arthroplasty in the United States from 2005 to 2030. J Bone Joint Surg.

[2] Pruzansky JS, Bronson MJ, Grelsamer RP, Strauss E, Moucha CS (2014). Prevalence of modifiable surgical site infection risk factors in hip and knee joint arthroplasty patients at an urban academic hospital. J Arthroplast.

[3] Marmor S, Kerroumi Y (2016). Patient-specific risk factors for infection in arthroplasty procedure. Orthop Traumatol Surg Res.

[4] Lee J, Singletary R, Schmader K, Anderson DJ, Bolognesi M, Kaye KS (2006). Surgical site infection in the elderly following orthopaedic surgery: risk factors and outcomes. J Bone Joint Surg.

[5] Pulido L, Ghanem E, Joshi A, Purtill JJ, Parvizi J (2008). Periprosthetic joint infection: the incidence, timing, and predisposing factors. Clin Orthop Relat Res.

[6] Ridgeway S, Wilson J, Charlet A, Kafatos G, Pearson A, Coello R (2005). Infection of the surgical site after arthroplasty of the hip. J Bone Joint Surg Br.

[7] Kalmeijer MD, van Nieuwland-Bollen E, Bogaers-Hofman D, de Baere GA (2000). Nasal carriage of Staphylococcus aureus is a major risk factor for surgical-site infections in orthopedic surgery. Infect Control Hosp Epidemiol.

[8] Cunningham DJ, Kavolus JJ, Bolognesi MP, Wellman SS, Seyler TM (2017). Specific infectious organisms associated with poor outcomes in treatment for hip periprosthetic infection. J Arthroplast.

[9] Wertheim HF, Vos MC, Ott A (2004). Risk and outcome of nosocomial *Staphylococcus aureus* bacteraemia in nasal carriers versus non-carriers. Lancet (London, England).

[10] Borthakur B, Kumar S, Talukdar M, Bidyananda A (2016). Surgical site infection in orthopaedics. Int J Orthop Sci.

[11] Hansen S, Schwab F, Asensio A (2010). Methicillin-resistant Staphylococcus aureus (MRSA) in Europe: which infection control measures are taken?. Infection.

[12] West SK, Plantenga MS, Strausbaugh LJ (2007). Use of decolonization to prevent staphylococcal infections in various healthcare settings: results of an emerging infections network survey. Infect Control Hosp Epidemiol.

[13] Rao N, Kim DH (2015). Perioperative risk factors and patient optimisation: risk assement and prevention. Let’s discuss surgical site infection ed.

[14] Wilcox MH, Hall J, Pike H (2003). Use of perioperative mupirocin to prevent methicillin-resistant Staphylococcus aureus (MRSA) orthopaedic surgical site infections. J Hosp Infect.

[15] Lee BY, Wiringa AE, Bailey RR (2010). The economic effect of screening orthopedic surgery patients preoperatively for methicillin-resistant Staphylococcus aureus. Infect Control Hosp Epidemiol.

[16] Rao N, Cannella B, Crossett LS, Yates AJ, McGough R (2008). A preoperative decolonization protocol for staphylococcus aureus prevents orthopaedic infections. Clin Orthop Relat Res.

[17] Bongartz T, Halligan CS, Osmon DR (2008). Incidence and risk factors of prosthetic joint infection after total hip or knee replacement in patients with rheumatoid arthritis. Arthritis Rheum.

[18] Goeb V, Ardizzone M, Arnaud L (2013). Recommendations for using TNFalpha antagonists and French clinical practice guidelines endorsed by the French National Authority for Health. Joint Bone Spine.

[19] Goodman SM, Springer B, Guyatt G (2017). 2017 American College of Rheumatology/American Association of hip and knee surgeons guideline for the perioperative management of antirheumatic medication in patients with rheumatic diseases undergoing elective total hip or total knee arthroplasty. J Arthroplast.

[20] Grijalva CG, Chen L, Delzell E (2011). Initiation of tumor necrosis factor-alpha antagonists and the risk of hospitalization for infection in patients with autoimmune diseases. J Am Med Assoc.

[21] Widdifield J, Bernatsky S, Paterson JM (2013). Serious infections in a population-based cohort of 86,039 seniors with rheumatoid arthritis. Arthritis Care Res.

[22] Bozic KJ, Lau E, Kurtz S, Ong K, Berry DJ (2012). Patient-related risk factors for postoperative mortality and periprosthetic joint infection in medicare patients undergoing TKA. Clin Orthop Relat Res.

[23] Korol E, Johnston K, Waser N (2013). A systematic review of risk factors associated with surgical site infections among surgical patients. PLoS One.

[24] Aggarwal VK, Tischler EH, Post ZD, Kane I, Orozco FR, Ong A (2013). Patients with atrial fibrillation undergoing total joint arthroplasty increase hospital burden. J Bone Joint Surg Am.

[25] Gill GS, Mills D, Joshi AB (2003). Mortality following primary total knee arthroplasty. J Bone Joint Surg Am.

[26] Han IH, Kim KS, Park HC (2009). Spinal surgery in patients with end-stage renal disease undergoing hemodialysis therapy. Spine.

[27] Parvizi J, Ghanem E, Joshi A, Sharkey PF, Hozack WJ, Rothman RH (2007). Does “excessive” anticoagulation predispose to periprosthetic infection?. J Arthroplast.

[28] Mraovic B, Suh D, Jacovides C, Parvizi J (2011). Perioperative hyperglycemia and postoperative infection after lower limb arthroplasty. J Diabetes Sci Technol.

[29] Zambouri A (2007). Preoperative evaluation and preparation for anesthesia and surgery. Hippokratia.

[30] Chen JH, Kuo FC, Wang JW (2014). Total knee arthroplasty in patients with dialysis: early complications and mortality. Biom J.

[31] McCleery MA, Leach WJ, Norwood T (2010). Rates of infection and revision in patients with renal disease undergoing total knee replacement in Scotland. J Bone Joint Surg Br.

[32] Fischer UM, Weissenberger WK, Warters RD, Geissler HJ, Allen SJ, Mehlhorn U (2002). Impact of cardiopulmonary bypass management on postcardiac surgery renal function. Perfusion.

[33] Cockcroft DW, Gault MH (1976). Prediction of creatinine clearance from serum creatinine. Nephron.

[34] Lieberman JR, Fuchs MD, Haas SB (1995). Hip arthroplasty in patients with chronic renal failure. J Arthroplast.

[35] Tannenbaum DA, Matthews LS, Grady-Benson JC (1997). Infection around joint replacements in patients who have a renal or liver transplantation. J Bone Joint Surg.

[36] Sakalkale DP, Hozack WJ, Rothman RH (1999). Total hip arthroplasty in patients on long-term renal dialysis. J Arthroplast.

[37] Lieu D, Harris IA, Naylor JM, Mittal R (2014). Review article: Total hip replacement in haemodialysis or renal transplant patients. J Orthop Surg (Hong Kong).

[38] Cavanaugh PK, Chen AF, Rasouli MR, Post ZD, Orozco FR, Ong AC (2016). Complications and mortality in chronic renal failure patients undergoing total joint arthroplasty: a comparison between dialysis and renal transplant patients. J Arthroplast.

[39] Shrader MW, Schall D, Parvizi J, McCarthy JT, Lewallen DG (2006). Total hip arthroplasty in patients with renal failure: a comparison between transplant and dialysis patients. J Arthroplast.

[40] Passalacqua JA, Wiland AM, Fink JC, Bartlett ST, Evans DA, Keay S (1999). Increased incidence of postoperative infections associated with peritoneal dialysis in renal transplant recipients. Transplantation.

[41] Hales CM, Carroll MD, Fryar CD, Ogden CL. Prevalence of obesity among adults and youth: United States, 2015-2016. NCHS Data Brief. 2017;(288):1–8. 29155689

[42] Yuan K, Chen H-L (2013). Obesity and surgical site infections risk in orthopedics: a meta-analysis. Int J Surg.

[43] Namba RS, Paxton L, Fithian DC, Stone ML (2005). Obesity and perioperative morbidity in total hip and total knee arthroplasty patients. J Arthroplast.

[44] Women M (2015). The impact of obesity on bone and joint health.

[45] Liabaud B, Patrick DA, Geller JA (2013). Higher body mass index leads to longer operative time in total knee arthroplasty. J Arthroplast.

[46] Falagas ME, Kompoti M (2006). Obesity and infection. Lancet Infect Dis.

[47] Burns JL, Mancoll JS, Phillips LG (2003). Impairments to wound healing. Clin Plast Surg.

[48] Yosipovitch G, DeVore A, Dawn A (2007). Obesity and the skin: skin physiology and skin manifestations of obesity. J Am Acad Dermatol.

[49] Markman B (1989). Anatomy and physiology of adipose tissue. Clin Plast Surg.

[50] Kawai K, Kageyama A, Tsumano T (2008). Effects of adiponectin on growth and differentiation of human keratinocytes--implication of impaired wound healing in diabetes. Biochem Biophys Res Commun.

[51] Cottam DR, Mattar SG, Barinas-Mitchell E (2004). The chronic inflammatory hypothesis for the morbidity associated with morbid obesity: implications and effects of weight loss. Obes Surg.

[52] Pierpont YN, Dinh TP, Salas RE (2014). Obesity and surgical wound healing: a current review. ISRN Obes.

[53] Winiarsky R, Barth P, Lotke P (1998). Total knee arthroplasty in morbidly obese patients. J Bone Joint Surg.

[54] O'Rourke RW, Kay T, Lyle EA (2006). Alterations in peripheral blood lymphocyte cytokine expression in obesity. Clin Exp Immunol.

[55] Motaghedi R, Bae JJ, Memtsoudis SG (2014). Association of obesity with inflammation and pain after total hip arthroplasty. Clin Orthop Relat Res.

[56] Mihalko WM, Bergin PF, Kelly FB, Canale ST (2014). Obesity, orthopaedics, and outcomes. J Am Acad Orthop Surg.

[57] Akiboye F, Rayman G (2017). Management of hyperglycemia and diabetes in orthopedic surgery. Curr Diab Rep.

[58] Martin ET, Kaye KS, Knott C (2016). Diabetes and risk of surgical site infection: a systematic review and meta-analysis. Infect Control Hosp Epidemiol.

[59] Onyekwelu I, Yakkanti R, Protzer L, Pinkston CM, Tucker C, Seligson D (2017). Surgical wound classification and surgical site infections in the orthopaedic patient. J Am Acad Orthop Surg Glob Res Rev.

[60] Olsen MA, Nepple JJ, Riew KD (2008). Risk factors for surgical site infection following orthopaedic spinal operations. J Bone Joint Surg Am.

[61] Marchant MH, Viens NA, Cook C, Vail TP, Bolognesi MP (2009). The impact of glycemic control and diabetes mellitus on perioperative outcomes after total joint arthroplasty. J Bone Joint Surg Am.

[62] Coords M, Breitbart E, Paglia D (2011). The effects of low-intensity pulsed ultrasound upon diabetic fracture healing. J Orthop Res.

[63] Wukich DK (2015). Diabetes and its negative impact on outcomes in orthopaedic surgery. World J Orthod.

[64] Lovecchio F, Beal M, Kwasny M, Manning D (2014). Do patients with insulin-dependent and noninsulin-dependent diabetes have different risks for complications after arthroplasty?. Clin Orthop Relat Res.

[65] Schipper ON, Jiang JJ, Chen L, Koh J, Toolan BC (2015). Effect of diabetes mellitus on perioperative complications and hospital outcomes after ankle arthrodesis and total ankle arthroplasty. Foot Ankle Int.

[66] Turina M, Fry DE, Polk HC (2005). Acute hyperglycemia and the innate immune system: clinical, cellular, and molecular aspects. Crit Care Med.

[67] Richards JE, Kauffmann RM, Zuckerman SL, Obremskey WT, May AK (2012). Relationship of hyperglycemia and surgical-site infection in orthopaedic surgery. J Bone Joint Surg Am.

[68] Stryker LS, Abdel MP, Morrey ME, Morrow MM, Kor DJ, Morrey BF (2013). Elevated postoperative blood glucose and preoperative hemoglobin A1C are associated with increased wound complications following total joint arthroplasty. J Bone Joint Surg Am.

[69] Kheir MM, Tan TL, Kheir M, Maltenfort MG, Chen AF (2018). Postoperative blood glucose levels predict infection after total joint arthroplasty. J Bone Joint Surg Am.

[70] Jamsen E, Nevalainen P, Kalliovalkama J, Moilanen T (2010). Preoperative hyperglycemia predicts infected total knee replacement. Eur J Intern Med.

[71] Sancheti PSA. Mannual of infection control in orthopaedic surgery. 1st ed: Jaypee Brothers Medical Publishers; 2015.

[72] Alamanda VK, Springer BD (2018). Perioperative and modifiable risk factors for periprosthetic joint infections (PJI) and recommended guidelines. Curr Rev Musculoskelet Med.

[73] Machino M, Yukawa Y, Ito K (2014). Risk factors for poor outcome of cervical laminoplasty for cervical spondylotic myelopathy in patients with diabetes. J Bone Joint Surg Am.

[74] Ljungqvist O, Soop M, Hedstrom M (2007). Why metabolism matters in elective orthopedic surgery: a review. Acta Orthop.

[75] Chatterjee R, Narayan KM, Lipscomb J, Phillips LS (2010). Screening adults for pre-diabetes and diabetes may be cost-saving. Diabetes Care.

[76] Chatterjee R, Narayan KMV, Lipscomb J (2013). Screening for diabetes and prediabetes should be cost-saving in patients at high risk. Diabetes Care.

[77] Umpierrez GE, Smiley D, Jacobs S (2011). Randomized study of basal-bolus insulin therapy in the inpatient management of patients with type 2 diabetes undergoing general surgery (RABBIT 2 surgery). Diabetes Care.

[78] Agos F, Shoda C, Bransford D (2014). Part II: managing perioperative hyperglycemia in total hip and knee replacement surgeries. Nurs Clin North Am.

[79] Cappellini MD, Motta I (2015). Anemia in clinical practice-definition and classification: does hemoglobin change with aging?. Semin Hematol.

[80] Goodnough LT, Vizmeg K, Sobecks R, Schwarz A, Soegiarso W (1992). Prevalence and classification of Anemia in elective orthopedic surgery patients: implications for blood conservation programs. Vox Sang.

[81] Dunne JR, Malone D, Tracy JK, Gannon C, Napolitano LM (2002). Perioperative anemia: an independent risk factor for infection, mortality, and resource utilization in surgery. J Surg Res.

[82] Greenky M, Gandhi K, Pulido L, Restrepo C, Parvizi J (2012). Preoperative anemia in total joint arthroplasty: is it associated with periprosthetic joint infection?. Clin Orthop Relat Res.

[83] Marik PE (2009). The hazards of blood transfusion. Br J Hosp Med.

[84] Bisbe E, Basora M, Colomina MJ, Spanish Best Practice in Peri-operative Anaemia Optimisation P (2017). Peri-operative treatment of anaemia in major orthopaedic surgery: a practical approach from Spain. Blood Transfus.

[85] Goodnough LT, Maniatis A, Earnshaw P (2011). Detection, evaluation, and management of preoperative anaemia in the elective orthopaedic surgical patient: NATA guidelines. Br J Anaesth.

[86] Theusinger OM, Kind SL, Seifert B, Borgeat L, Gerber C, Spahn DR (2014). Patient blood management in orthopaedic surgery: a four-year follow-up of transfusion requirements and blood loss from 2008 to 2011 at the Balgrist University Hospital in Zurich, Switzerland. Blood Transfus.

[87] Goodnough LT, Skikne B, Brugnara C (2000). Erythropoietin, iron, and erythropoiesis. Blood.

[88] Bisbe E, García-Erce JA, Díez-Lobo AI, Muñoz M (2011). A multicentre comparative study on the efficacy of intravenous ferric carboxymaltose and iron sucrose for correcting preoperative anaemia in patients undergoing major elective surgery. Br J Anaesth.

[89] Munoz M, Garcia-Erce JA, Diez-Lobo AI, Campos A, Sebastianes C, Bisbe E (2009). Usefulness of the administration of intravenous iron sucrose for the correction of preoperative anemia in major surgery patients. Med Clin.

[90] Serrano-Trenas JA, Ugalde PF, Cabello LM, Chofles LC, Lázaro PS, Benítez PC (2011). Role of perioperative intravenous iron therapy in elderly hip fracture patients: a single-center randomized controlled trial. Transfusion.

[91] Johnson OC, Chebli C, Aboulafia AJ (2003). Epoetin alfa. J Am Acad Orthop Surg.

[92] Foley RN, Curtis BM, Parfrey PS (2009). Erythropoietin therapy, hemoglobin targets, and quality of life in healthy hemodialysis patients: a randomized trial. Clin J Am Soc Nephrol.

[93] Moonen AFCM, Thomassen BJW, Knoors NT, van Os JJ, Verburg AD, Pilot P (2008). Pre-operative injections of epoetin-α versus post-operative retransfusion of autologous shed blood in total hip and knee replacement. J Bone Joint Surg.

[94] Baldo BA (2014). Side effects of cytokines approved for therapy. Drug Saf.

[95] Kotzé A, Carter LA, Scally AJ (2012). Effect of a patient blood management programme on preoperative anaemia, transfusion rate, and outcome after primary hip or knee arthroplasty: a quality improvement cycle. Br J Anaesth.

[96] Freedman J (2014). The ONTraC Ontario program in blood conservation. Transfus Apher Sci.

[97] Yuwen P, Chen W, Lv H (2017). Albumin and surgical site infection risk in orthopaedics: a meta-analysis. BMC Surg.

[98] Hill G, Blackett R, Pickford I (1977). Malnutrition in surgical patients. An unrecognised problem. Lancet.

[99] Lesourd B, Mazari L (1999). Nutrition and immunity in the elderly. Proc Nutr Soc.

[100] Huang R, Greenky M, Kerr GJ, Austin MS, Parvizi J (2013). The effect of malnutrition on patients undergoing elective joint arthroplasty. J Arthroplast.

[101] Dickhaut S, DeLee JC, Page CP (1984). Nutritional status: importance in predicting wound-healing after amputation. J Bone Joint Surg.

[102] Cross MB, Yi P, Thomas CF, Garcia J, Della Valle CJ (2014). Evaluation of malnutrition in orthopaedic surgery. J Am Acad Orthop Surg.

[103] Walls JD, Abraham D, Nelson CL, Kamath AF, Elkassabany NM, Liu J (2015). Hypoalbuminemia more than morbid obesity is an independent predictor of complications after total hip arthroplasty. J Arthroplast.

[104] Mullen JL, Gertner MH, Buzby GP, Goodhart GL, Rosato EF (1979). Implications of malnutrition in the surgical patient. Arch Surg.

[105] Gherini S, Vaughn BK, Lombardi AV, Mallory TH (1993). Delayed wound healing and nutritional deficiencies after total hip arthroplasty. Clin Orthop Relat Res.

[106] Kuzuya M, Kanda S, Koike T, Suzuki Y, Iguchi A (2005). Lack of correlation between total lymphocyte count and nutritional status in the elderly. Clin Nutr.

[107] Jensen JE, Jensen TG, Smith TK, Johnston DA, Dudrick SJ (1982). Nutrition in orthopaedic surgery. J Bone Joint Surg.

[108] Jones RE, Russell RD, Huo MH (2013). Wound healing in total joint replacement. Bone Joint J.

[109] Greene KA, Wilde AH, Stulberg BN (1991). Preoperative nutritional status of total joint patients. Relationship to postoperative wound complications. J Arthroplasty.

[110] Di Monaco M, Di Monaco R, Manca M, Cavanna A (2002). Positive association between total lymphocyte count and femur bone mineral density in hip-fractured women. Gerontology.

[111] Hewison M (2010). Vitamin D and the immune system: new perspectives on an old theme. Endocrinol Metab Clin N Am.

[112] Maier GS, Horas K, Seeger JB, Roth KE, Kurth AA, Maus U (2014). Is there an association between periprosthetic joint infection and low vitamin D levels?. Int Orthop.

[113] West MA, Wischmeyer PE, Grocott MPW (2017). Prehabilitation and nutritional support to improve perioperative outcomes. Curr Anesthesiol Rep.

[114] Durand F, Berthelot P, Cazorla C, Farizon F, Lucht F (2013). Smoking is a risk factor of organ/space surgical site infection in orthopaedic surgery with implant materials. Int Orthop.

[115] Williams G, Daly M, Proude EM (2008). The influence of alcohol and tobacco use in orthopaedic inpatients on complications of surgery. Drug Alcohol Rev.

[116] Best MJ, Buller LT, Gosthe RG, Klika AK, Barsoum WK (2015). Alcohol misuse is an independent risk factor for poorer postoperative outcomes following primary total hip and total knee arthroplasty. J Arthroplasty.

[117] Jamal A, Phillips E, Gentzke AS (2018). Current cigarette smoking among adults — United States, 2016. MMWR Morb Mortal Wkly Rep.

[118] Singh JA, Schleck C, Harmsen WS, Jacob AK, Warner DO, Lewallen DG (2015). Current tobacco use is associated with higher rates of implant revision and deep infection after total hip or knee arthroplasty: a prospective cohort study. BMC Med.

[119] Rodgman A, Smith CJ, Perfetti TA (2000). The composition of cigarette smoke: a retrospective, with emphasis on polycyclic components. Hum Exp Toxicol.

[120] Gullihorn L, Karpman R, Lippiello L (2005). Differential effects of nicotine and smoke condensate on bone cell metabolic activity. J Orthop Trauma.

[121] Rottenstein H, Peirce G, Russ E, Felder D, Montgomery H (1960). Influence of nicotine on the blood flow of resting skeletal muscle and of the digits in normal subjects. Ann N Y Acad Sci.

[122] Guo S, Dipietro LA (2010). Factors affecting wound healing. J Dent Res.

[123] Sorensen LT (2012). Wound healing and infection in surgery: the pathophysiological impact of smoking, smoking cessation, and nicotine replacement therapy: a systematic review. Ann Surg.

[124] National Center for Chronic Disease Prevention and Health Promotion (US) Office on Smoking and Health. The health consequences of smoking—50 years of progress: a report of the surgeon general. Ctr Dis Control Prev (US). 2014. 24455788

[125] Moller AM, Pedersen T, Villebro N, Munksgaard A (2003). Effect of smoking on early complications after elective orthopaedic surgery. J Bone Joint Surg.

[126] Teng S, Yi C, Krettek C, Jagodzinski M (2015). Smoking and risk of prosthesis-related complications after total hip arthroplasty: a meta-analysis of cohort studies. PLoS One.

[127] Peersman G, Laskin R, Davis J, Peterson M (2001). Infection in total knee replacement: a retrospective review of 6489 total knee replacements. Clin Orthop Relat Res.

[128] Kong L, Liu Z, Meng F, Shen Y (2016). Smoking and risk of surgical site infection after spinal surgery: a systematic review and meta-analysis. Surg Infect.

[129] Scolaro JA, Schenker ML, Yannascoli S, Baldwin K, Mehta S, Ahn J (2014). Cigarette smoking increases complications following fracture: a systematic review. J Bone Joint Surg.

[130] Moucha CS, Clyburn T, Evans RP, Prokuski L (2011). Modifiable risk factors for surgical site infection. J Bone Joint Surg.

[131] Thomsen T, Villebro N, Moller AM (2010). Interventions for preoperative smoking cessation. Cochrane Database Syst Rev.

[132] Singh JA (2011). Smoking and outcomes after knee and hip arthroplasty: a systematic review. J Rheumatol.

[133] Mills E, Eyawo O, Lockhart I, Kelly S, Wu P, Ebbert JO (2011). Smoking cessation reduces postoperative complications: a systematic review and meta-analysis. Am J Med.

[134] Nolan MB, Martin DP, Thompson R, Schroeder DR, Hanson AC, Warner DO (2017). Association between smoking status, preoperative exhaled carbon monoxide levels, and postoperative surgical site infection in patients undergoing elective surgery. Smoking status and surgical site infection after elective surgery. JAMA Surg.

[135] Lindström D, Azodi OS, Wladis A (2008). Effects of a perioperative smoking cessation intervention on postoperative complications: a randomized trial. Ann Surg.

[136] (CDC) CfDCaP (2011). Quitting smoking among adults--United States, 2001-2010. MMWR Morb Mortal Wkly Rep.

[137] Pirkle JL, Flegal KM, Bernert JT, Brody DJ, Etzel RA, Maurer KR (1996). Exposure of the US population to environmental tobacco smoke: the third National Health and nutrition examination survey, 1988 to 1991. J Am Med Assoc.

[138] Cromwell J, Bartosch WJ, Fiore MC, Hasselblad V, Baker T (1997). Cost-effectiveness of the clinical practice recommendations in the AHCPR guideline for smoking cessation. J Am Med Assoc.

[139] Dowd B, Li C-h, Swenson T, Coulam R, Levy J (2014). Medicare’s physician quality reporting system (PQRS): quality measurement and beneficiary attribution. Medicare Medicaid Res Rev.

[140] Thomsen T, Tonnesen H, Moller AM (2009). Effect of preoperative smoking cessation interventions on postoperative complications and smoking cessation. Br J Surg.

[141] Moller AM, Villebro N, Pedersen T, Tonnesen H (2002). Effect of preoperative smoking intervention on postoperative complications: a randomised clinical trial. Lancet.

[142] Lowry F (2011). Alcohol linked to complications after joint surgery.

[143] Tonnesen H (2003). Alcohol abuse and postoperative morbidity. Dan Med Bull.

[144] Spies CD, von Dossow V, Eggers V (2004). Altered cell-mediated immunity and increased postoperative infection rate in long-term alcoholic patients. Anesthesiology.

[145] Rotevatn TA, Bøggild H, Olesen CR (2017). Alcohol consumption and the risk of postoperative mortality and morbidity after primary hip or knee arthroplasty – a register-based cohort study. PLoS One.

[146] Oppedal K, Moller AM, Pedersen B, Tonnesen H. Preoperative alcohol cessation prior to elective surgery. Cochrane Database Syst Rev. 2012;(7):Cd008343. 10.1002/14651858.CD008343.pub2. 10.1002/14651858.CD008343.pub2 22786514

[147] Amato L, Minozzi S, Vecchi S, Davoli M. Benzodiazepines for alcohol withdrawal. Cochrane Database Syst Rev. 2010;(3):Cd005063. 10.1002/14651858.CD005063.pub3 PMC12414628 20238336

[148] Rosner S, Hackl-Herrwerth A, Leucht S, Lehert P, Vecchi S, Soyka M. Acamprosate for alcohol dependence. Cochrane Database Syst Rev. 2010;(9):Cd004332. 10.1002/14651858.CD004332.pub2 PMC12147086 20824837

[149] Rösner S, Hackl-Herrwerth A, Leucht S, Vecchi S, Srisurapanont M, Soyka M. Opioid antagonists for alcohol dependence. Cochrane Database Syst Rev. 2010;12. 10.1002/14651858.CD001867.pub2. 10.1002/14651858.CD001867.pub3 PMC13590218 21154349

[150] Jorgensen CH, Pedersen B, Tonnesen H (2011). The efficacy of disulfiram for the treatment of alcohol use disorder. Alcohol Clin Exp Res.

[151] Solberg LI, Maciosek MV, Edwards NM (2008). Primary care intervention to reduce alcohol misuse: ranking its health impact and cost effectiveness. Am J Prev Med.

[152] Ghoneim MM, O'Hara MW (2016). Depression and postoperative complications: an overview. BMC Surg.

[153] Ali A, Lindstrand A, Sundberg M, Flivik G (2017). Preoperative anxiety and depression correlate with dissatisfaction after total knee arthroplasty: a prospective longitudinal cohort study of 186 patients, with 4-year follow-up. J Arthroplast.

[154] Schwartz FH, Lange J (2017). Factors that affect outcome following total joint arthroplasty: a review of the recent literature. Curr Rev Musculoskelet Med.

[155] Rasouli MR, Menendez ME, Sayadipour A, Purtill JJ, Parvizi J (2016). Direct cost and complications associated with total joint arthroplasty in patients with preoperative anxiety and depression. J Arthroplasty.

[156] Kunutsor SK, Whitehouse MR, Blom AW, Beswick AD, Team I (2016). Patient-related risk factors for periprosthetic joint infection after total joint arthroplasty: a systematic review and meta-analysis. PLoS One.

[157] Stein M, Keller SE, Schleifer SJ (1988). Immune system. Relationship to anxiety disorders. Psychiatr Clin North Am.

[158] Elenkov IJ (2002). Systemic stress-induced Th2 shift and its clinical implications. Int Rev Neurobiol.

[159] Molendijk ML, Spinhoven P, Polak M, Bus BAA, Penninx BWJH, Elzinga BM (2013). Serum BDNF concentrations as peripheral manifestations of depression: evidence from a systematic review and meta-analyses on 179 associations (*N*=9484). Mol Psychiatry.

[160] Bufalino C, Hepgul N, Aguglia E, Pariante CM (2013). The role of immune genes in the association between depression and inflammation: a review of recent clinical studies. Brain Behav Immun.

[161] Alattas SA, Smith T, Bhatti M, Wilson-Nunn D, Donell S (2017). Greater pre-operative anxiety, pain and poorer function predict a worse outcome of a total knee arthroplasty. Knee Surg Sports Traumatol Arthrosc.

[162] Nickinson RSJ, Board TN, Kay PR (2009). Post-operative anxiety and depression levels in orthopaedic surgery: a study of 56 patients undergoing hip or knee arthroplasty. J Eval Clin Pract.

